# Machine-learning classification of children and adolescents with ASD using resting-state frequency-specific intrinsic activity

**DOI:** 10.3389/fnins.2026.1892288

**Published:** 2026-08-07

**Authors:** Qi Huang, Sisi Jiang, Cheng Luo, Dezhong Yao, Bharat B. Biswal, Benjamin Klugah-Brown

**Affiliations:** 1 MOE Key Laboratory for Neuroinformation, Center for Information in Medicine, School of Life Science and Technology, University of Electronic Science and Technology of China, Chengdu, China; 2 High-Field Magnetic Resonance Brain Imaging Key Laboratory of Sichuan Province, Center for Information in Medicine, School of Life Science and Technology, University of Electronic Science and Technology of China, Chengdu, China; 3 China-Cuba Belt and Road Joint Laboratory on Neurotechnology and Brain-Apparatus Communication, School of Life Science and Technology, University of Electronic Science and Technology of China, Chengdu, China; 4 Department of Biomedical Engineering, New Jersey Institute of Technology, Newark, NJ, United States

**Keywords:** age-defined developmental stage, ALFF, autism spectrum disorder, CovBat, frequency-specific analysis, multivariate pattern analysis, ReHo, resting-state fMRI

## Abstract

**Background:**

Autism spectrum disorder (ASD) is characterized by heterogeneous developmental trajectories, yet it remains unclear whether frequency-specific resting-state functional magnetic resonance imaging (rs-fMRI) features can distinguish age-defined developmental stages within the condition.

**Methods:**

We analyzed rs-fMRI data from 251 participants with ASD, comprising 146 children and 105 adolescents aggregated from ten sites in the Autism Brain Imaging Data Exchange (ABIDE). ALFF and ReHo were computed across three frequency bands: Conventional (0.01–0.08 Hz), slow-4 (0.027–0.073 Hz), and slow-5 (0.01–0.027 Hz). Region-of-interest features were extracted using the 246-region Brainnetome Atlas. To ensure rigorous generalization, participants were divided into a stratified training set (80%, *n* = 200) and a held-out test set (20%, *n* = 51), with stratification based on the child–adolescent group label and a fixed random seed of 42. CovBat harmonization parameters, feature-scaling parameters, LASSO feature selection, and classifier hyperparameters were estimated using the training data only and subsequently applied to the held-out test data. Final model performance was evaluated once on the held-out test set. Performance was evaluated using Logistic Regression (LR), Support Vector Machine, and Random Forest classifiers, with Shapley Additive Explanations (SHAP) used to characterized interpret feature contributions.

**Results:**

The slow-4 and Conventional-band features showed higher held-out ASD test-set performance than slow-5 features. The best single-metric model by area under the receiver operating characteristic curve (AUC) was slow-4 ReHo Logistic Regression, which achieved an AUC of 0.811 and accuracy of 0.745. The exploratory combined model using slow-4 ALFF and ReHo features achieved the highest overall AUC of 0.819 (accuracy = 0.725). SHAP analysis identified distributed model-contributing regions in the slow-4 ReHo model, including the inferior parietal lobule, lateral occipital cortex, middle and inferior frontal gyri, basal ganglia, and thalamus.

**Conclusion:**

Frequency-specific resting-state features, particularly local synchronization in the slow-4 band, capture developmental-stage-related variation within ASD. The involvement of frontoparietal, visual, and subcortical networks suggests that developmental heterogeneity in ASD is supported by distributed reorganization of intrinsic brain activity. These findings highlight the potential of frequency-specific rs-fMRI metrics as candidate markers for characterizing neurodevelopmental stages in ASD, warranting further validation in longitudinal and independent cohorts.

## 1 Introduction

Autism spectrum disorder (ASD) is a highly heterogeneous neurodevelopmental condition characterized by persistent social communication impairments and restricted, repetitive behaviors (Geschwind and Levitt, 2007). The clinical presentation of ASD encompasses a striking diversity in cognitive profiles, symptom severity, and developmental trajectories (Lord et al., 2018). This heterogeneity is associated with neurobiological variation and may reflect diverse developmental pathways (Abrams et al., 2013). Previous studies have suggested that such heterogeneity is related to atypical neurodevelopmental trajectories occurring across critical periods of brain maturation. For instance, functional brain abnormalities in ASD exhibit dynamic, age-dependent patterns from childhood to adolescence (Li et al., 2025). Specifically, a period of dysregulated maturation occurs during preadolescence in the autistic brain (He et al., 2020).

Neurotypical development involves profound reorganization during childhood and adolescence, including synaptic pruning and network integration (Casey et al., 2025). These processes refine functional connectivity in systems involved in social cognition, emotion regulation and executive control (Casey et al., 2005; Crone and Dahl, 2012). Adolescence represents a critical period of neural reconfiguration, marked by shifts in cortical--subcortical balance (Casey et al., 2019), reorganization of default mode and frontoparietal networks (Sherman et al., 2014), and heightened plasticity in social circuits. In ASD, developmental trajectories may deviate from typical patterns, with altered timing of network maturation potentially contributing to the substantial heterogeneity observed across individuals with ASD (Guo et al., 2017). Yet, few studies have directly examined within-ASD developmental differences, especially those distinguishing childhood from adolescence.

Resting-state functional magnetic resonance imaging (rs-fMRI) captures spontaneous fluctuations in the BOLD signal to investigate intrinsic brain organization (Biswal et al., 1995). Two complementary metrics, Amplitude of Low-Frequency Fluctuations (ALFF) and Regional Homogeneity (ReHo), are particularly informative in ASD research (Zang et al., 2004; Yang et al., 2007). ALFF reflects the strength of local neural activity, while ReHo indexes local functional coherence. Alterations in both measures have been reported in the social brain (Paakki et al., 2010), limbic system (Schumann et al., 2011), and the default mode network (Padmanabhan et al., 2017). These findings have been interpreted as alterations in processes related to socio-emotional and cognitive functioning (Uddin et al., 2013).

The BOLD signal exhibits frequency-specific organization, with distinct low-frequency oscillations reflecting different aspects of intrinsic brain activity and functional organization. The slow-5 (0.01–0.027 Hz) band indexes long-range integrative activity, whereas the slow-4 (0.027–0.073 Hz) and Conventional (0.01–0.08 Hz) bands are more sensitive to regional fluctuations associated with sensory and cognitive processing (Zuo et al., 2010). Conversely, higher-frequency bands like slow-2 and slow-3 are often dominated by physiological noise (Bianciardi et al., 2009), while the slow-6 band is susceptible to very low-frequency drifts (Cordes et al., 2001). Focusing on slow-4 and slow-5 effectively captures relevant oscillations while mitigating confounding artifacts. Frequency-dependent analysis suggests that distinct bands may capture complementary aspects of brain function (Deng et al., 2023). In ASD, age-related effects may preferentially emerge in higher-frequency bands like slow-4 (Mei et al., 2022). Nevertheless, frequency-dependent maturation across the childhood--adolescence transition remains underexplored.

Machine learning (ML) offers tools to decode complex neural patterns beyond univariate analyses. By integrating multivariate relationships, ML models can identify multivariate patterns associated with developmental stages. ML has been applied to distinguish ASD from typical development or to predict behavioral phenotypes (Heinsfeld et al., 2018). However, multi-site datasets face challenges due to inter-site variability, which can reduce model generalizability (Pomponio et al., 2020).

To overcome this, harmonization techniques such as ComBat and CovBat correct for site-related variability in the mean, variance, and covariance of imaging features (Fortin et al., 2018; Chen et al., 2022). These tools, combined with robust ML frameworks, enable large-scale analyses across datasets like the Autism Brain Imaging Data Exchange (ABIDE) (Di Martino et al., 2013). Simultaneously, explainable ML methods like Shapley Additive Explanations (SHAP) can summarize which features contribute to model predictions (Lundberg and Lee, 2017).

The present study aimed to examine whether frequency-specific resting-state ALFF and ReHo features contain multivariate information associated with age-defined developmental stage within ASD. We analyzed harmonized multi-site rs-fMRI data from individuals with ASD aggregated across ten ABIDE sites. Participants were stratified into children aged ≥2 and ≤12 years and adolescents aged >12 and ≤21 years, with the developmental-stage grouping informed by pediatric age-stage definitions. ALFF and ReHo features were computed across three frequency bands: Conventional, slow-4, and slow-5. Following train-test partitioning and CovBat harmonization, LASSO feature selection and multiple supervised classifiers were used to evaluate developmental-stage-related multivariate patterns. SHAP analysis was then applied to summarize brain regions contributing to the final trained models.

We hypothesized that: (1) frequency-specific ALFF and ReHo features would contain above-chance information for distinguishing age-defined children and adolescents within ASD; (2) Conventional and slow-4 frequency bands would show stronger observed discriminative performance than the slow-5 band, consistent with prior evidence of frequency-specific age-related changes in spontaneous brain activity; and (3) model-contributing features would involve distributed regions implicated in social, limbic, and executive-control processes, providing anatomical context for developmental-stage-related multivariate patterns rather than direct evidence of causal or ASD-specific maturation effects.

## 2 Materials and methods

### 2.1 Participants

Resting-state fMRI and phenotypic data were obtained from the Autism Brain Imaging Data Exchange I and II repositories (ABIDE I/II) (Di Martino et al., 2017). The primary analysis included 251 individuals with a clinical diagnosis of autism spectrum disorder (ASD), as recorded in the ABIDE phenotypic files. Participants ranged from 2 to 21 years of age and were stratified into two non-overlapping developmental groups: children aged > = 2 and <=12 years (n = 146) and adolescents aged >12 and <=21 years (n = 105). This developmental-stage grouping was informed by pediatric age-stage definitions from the American Academy of Pediatrics (Hardin and Hackell, 2017).

Participants were included if they had: (1) a clinical diagnosis of ASD according to the original ABIDE site-specific diagnostic procedures; and (2) complete resting-state fMRI, demographic, and imaging quality-control data. Participants were excluded if they showed excessive head motion, defined as translational displacement >2 mm or rotational displacement >2°, mean framewise displacement (FD) > 0.2 mm, incomplete demographic information, or incomplete imaging-derived feature data.

Before CovBat harmonization or any machine-learning procedure, the 251 participants with ASD were divided into a training set and a held-out test set using a fixed 80:20 split. Stratification was performed according to the developmental-stage group label to preserve the relative proportions of children and adolescents in both subsets. A fixed random seed of 42 was used to ensure reproducibility. The resulting training set included 200 participants, comprising 116 children and 84 adolescents, whereas the held-out test set included 51 participants, comprising 30 children and 21 adolescents. Participant identifiers assigned to the held-out test set were excluded from all estimation of harmonization parameters, feature-scaling parameters, feature-selection parameters, and classifier hyperparameters.

A separate typically developing (TD) control sample was included only for exploratory whole-brain control analyses. The TD sample included 325 participants, comprising 202 children aged ≥2 and ≤12 years and 123 adolescents aged >12 and ≤21 years. The same developmental-stage definitions were applied to the TD sample. TD participants were not used for ASD model training, feature selection, hyperparameter optimization, harmonization parameter estimation, model interpretation, or held-out ASD test evaluation. Instead, the TD analysis was conducted independently as an exploratory contextual analysis to assess whether corrected child-adolescent differences were observed outside the ASD machine-learning framework.

### 2.2 fMRI data preprocessing

The rs-fMRI data were preprocessed using the Data Processing & Analysis for Resting-State Brain Imaging (DPARSF (Yan and Zang, 2010), V4.5) toolbox, following a standardized pipeline. The initial preprocessing steps common to all analyses included: (1) removal of the first 10 temporal volumes to allow for signal stabilization; (2) slice-timing correction; (3) head motion correction via realignment; (4) co-registration of the functional images to the individual’s T1-weighted structural image; (5) spatial normalization to the Montreal Neurological Institute (MNI) standard space with resampling to 3 × 3 × 3 mm^3^ voxels; (6) nuisance covariate regression, including the Friston 24-parameter motion model, as well as signals from white matter and cerebrospinal fluid (CSF); and (7) linear detrending.

Two key rs-fMRI metrics were computed for each participant across three distinct frequency bands:

1 Regional Homogeneity (ReHo): The analysis was performed on the preprocessed data without spatial smoothing. A Gaussian kernel of 6 mm full-width at half-maximum (FWHM) was applied for spatial smoothing only after the ReHo maps were computed. The ReHo was used to evaluate the local synchronization of spontaneous neural activity. The method is based on the assumption that functionally related regions exhibit temporally similar BOLD signal fluctuations. For each voxel, ReHo was calculated by assessing the similarity of its time series with those of its neighboring voxels using Kendall’s coefficient of concordance (KCC).

Let K denote the number of neighboring voxels (including the center voxel, typically K = 27) and n the number of time points in the fMRI time series. The KCC is defined in Equation 1:


$$W=\frac{12\sum_{t=1}^{n}{\left(R_{t}-\bar{R}\right)}^{2}}{K^{2}(n^{3}-n)}$$ (1)

Where W is the Kendall’s W (i.e., the ReHo value), $R_{t}$ is the sum of ranks for the K voxels at time point t , $\bar{R}$ is the mean of $R_{t}$ over all time points.

2 Amplitude of Low-Frequency Fluctuation (ALFF): Temporal band-pass filtering was applied prior to ALFF calculation (Yu-Feng et al., 2007). The data were then spatially smoothed with a 6 mm FWHM Gaussian kernel before feature extraction. The ALFF quantifies the amplitude of spontaneous neural activity in the low-frequency range of the BOLD signal. As shown in Equation 2, ALFF is calculated by first applying Fast Fourier Transform (FFT) to obtain the power spectrum and then computing the square root of the average power within a specified frequency range:

$$\mathrm{ALF}F_{i}=\frac{\sum_{f=f_{\min}}^{f_{\max}}\sqrt{P_{i}(f)}}{N_{f}}$$ (2)

Where $P_{i}(f)$ is the power spectral density at frequency f for voxel i and $N_{f}$ is the number of frequencies in the band. This reflects the intensity of low-frequency oscillations. The analyses were conducted in three frequency bands: Conventional band: 0.01–0.08 Hz, slow-4 band: 0.027–0.073 Hz, slow-5 band: 0.01–0.027 Hz. For each participant and each frequency band, the resulting voxel-wise ReHo and ALFF maps were standardized by converting them to Z-scores.

### 2.3 Data harmonization and feature selection

To account for site-related variability in the multisite neuroimaging data, we applied CovBat harmonization, a covariance-aware extension of the ComBat framework that adjusts site-related differences in feature means, variances, and covariance structure (Chen et al., 2022), as implemented in the DPABI Harmonization toolbox (Wang et al., 2024). The ASD cohort was divided into training and held-out test sets before CovBat harmonization. CovBat parameters were estimated using the training set only and were subsequently applied to the held-out test features without re-estimation. The held-out test set was therefore not used to estimate any harmonization parameters. ALFF and ReHo datasets were harmonized separately for each of the Conventional, slow-4, and slow-5 frequency bands. Acquisition site was treated as the batch variable, with age, sex, and mean framewise displacement included as covariates in the CovBat model.

For feature definition, we employed the Brainnetome Atlas (BNA), a connectivity-based parcellation derived from diffusion MRI and functional MRI data (Fan et al., 2016). The atlas subdivides the human brain into 246 fine-grained ROI (210 cortical and 36 subcortical), each annotated with rich information on anatomical location, connectivity profile, and functional annotation. The mean value of each imaging metric within these ROIs was extracted, yielding an initial feature set of 246 region-wise features. Following CovBat harmonization, ROI features were standardized using means and standard deviations estimated exclusively from the harmonized training data. The same training-derived scaling parameters were then applied unchanged to the held-out test data. No scaling parameter was estimated or updated using the held-out observations.

Feature selection was subsequently performed using the Least Absolute Shrinkage and Selection Operator (LASSO) within the training set (Tibshirani, 1996). The LASSO regularization parameter was selected using stratified 5-fold cross-validation applied only to the training data. Features with non-zero coefficients in the final training-set LASSO model were retained. The resulting feature mask was then applied unchanged to both the training and held-out test datasets. Thus, the held-out test labels and feature values were not used to determine the selected features.

### 2.4 Machine learning classification and model evaluation

Three supervised machine-learning classifiers were implemented using the LASSO-selected ROI-level features: support vector machine (SVM) with a radial basis function (RBF) kernel (Boser et al., 1992), Random Forest (RF) (Breiman, 2001), and Logistic Regression (LR) (LaValley, 2008). These classifiers were selected to represent complementary modeling strategies: SVM for capturing nonlinear decision boundaries, RF for ensemble-based tree classification, and LR as a parsimonious linear model.

Classification analyses were performed separately for each frequency-specific ALFF and ReHo feature set. In addition, exploratory combined-feature analyses were conducted by concatenating ALFF and ReHo features within each frequency band. This design allowed us to evaluate the frequency-specific and metric-specific contributions of regional activity amplitude and local functional synchronization to developmental-stage classification within ASD.

Classifier hyperparameters were optimized using stratified 5-fold cross-validation within the training set. The cross-validation folds were stratified by developmental-stage group label. For each classifier and feature set, the hyperparameter configuration with the highest mean cross-validated AUC was selected. The optimized classifier was then refitted using the complete training set and evaluated once on the held-out test set. The held-out test data were not used for hyperparameter selection, model fitting, or decision-threshold optimization.

The SVM classifier was implemented with an RBF kernel to capture nonlinear relationships in the selected feature space. Hyperparameter optimization was performed using grid search with stratified 5-fold cross-validation within the training set (Chang and Lin, 2011). The SVM grid searched C values of 0.1, 1, and 10 and gamma values of scale, 0.01, and 0.001. The optimal hyperparameter combination was selected according to the highest mean cross-validated AUC. The optimized SVM model was then refitted on the full training set and evaluated once on the independent held-out test set.

The RF classifier was implemented as an ensemble of decision trees with a fixed random seed to ensure reproducibility. Class-balanced weighting was used to reduce the influence of group imbalance. Hyperparameter optimization was performed using grid search with stratified 5-fold cross-validation within the training set. The number of trees was fixed at 300, while the maximum tree depth was searched over None, 5, and 10, and the minimum number of samples per leaf was searched over 1, 2, and 4. The best RF configuration was selected based on mean cross-validated AUC, refitted on the full training set, and evaluated on the held-out test set.

LR was included as a linear classifier to provide a parsimonious benchmark against the nonlinear and ensemble-based models. The LR model used L2 regularization, the liblinear solver, class-balanced weighting, and a maximum of 5,000 iterations to reduce overfitting, support convergence, and mitigate the influence of class imbalance. Feature standardization was performed within the model pipeline, with scaling parameters estimated from the training data only. Hyperparameter optimization was performed using grid search with stratified 5-fold cross-validation within the training set. The inverse regularization strength C was searched over 0.01, 0.1, 1, 10, and 100. The optimal LR model was selected according to mean cross-validated AUC, refitted on the full training set, and evaluated on the independent held-out test set.

### 2.5 Model evaluation strategy

Model development followed a fixed held-out evaluation design. First, the full ASD cohort was divided into a stratified training set (*n* = 200) and a held-out test set (*n* = 51) using an 80:20 split and a fixed random seed of 42. All subsequent data-dependent parameters, including CovBat harmonization parameters, feature-scaling statistics, LASSO regularization and feature selection, and classifier hyperparameters, were estimated using the training set only. Training-set cross-validation was used for model development and hyperparameter optimization. After the complete pipeline had been finalized, the training-derived transformations were applied to the held-out test set, and final performance was evaluated once. Held-out test-set metrics were treated as the primary internal estimates of generalization.

### 2.6 Performance metrics

Classification performance was assessed using accuracy, sensitivity, specificity, and area under the receiver operating characteristic curve (AUC) (Fawcett, 2006). Predicted probabilities were used to compute AUC. In the present coding scheme, children were labeled as 0 and adolescents were labeled as 1. Therefore, sensitivity represented the adolescent identification rate, whereas specificity represented the child identification rate. Ninety-five percent confidence intervals (95% CIs) for the primary performance metrics were estimated by bootstrap resampling of the predictions on the independent held-out test set using 1,000 bootstrap iterations (Paraschakis et al., 2024). The independent held-out test-set metrics were reported as the primary model performance estimates. In summary, the held-out test set remained isolated from CovBat parameter estimation, feature scaling, LASSO feature selection, hyperparameter optimization, and model fitting. The test set was accessed only after the corresponding training pipeline had been finalized.

### 2.7 Feature contribution analysis

To interpret the contribution of individual features during the classification process across the three frequency bands, we employed Shapley Additive Explanations (SHAP) analysis. SHAP is a unified framework based on cooperative game theory that assigns each feature an importance value, called the Shapley value, representing its contribution to the prediction for each sample.

**Figure 1:**
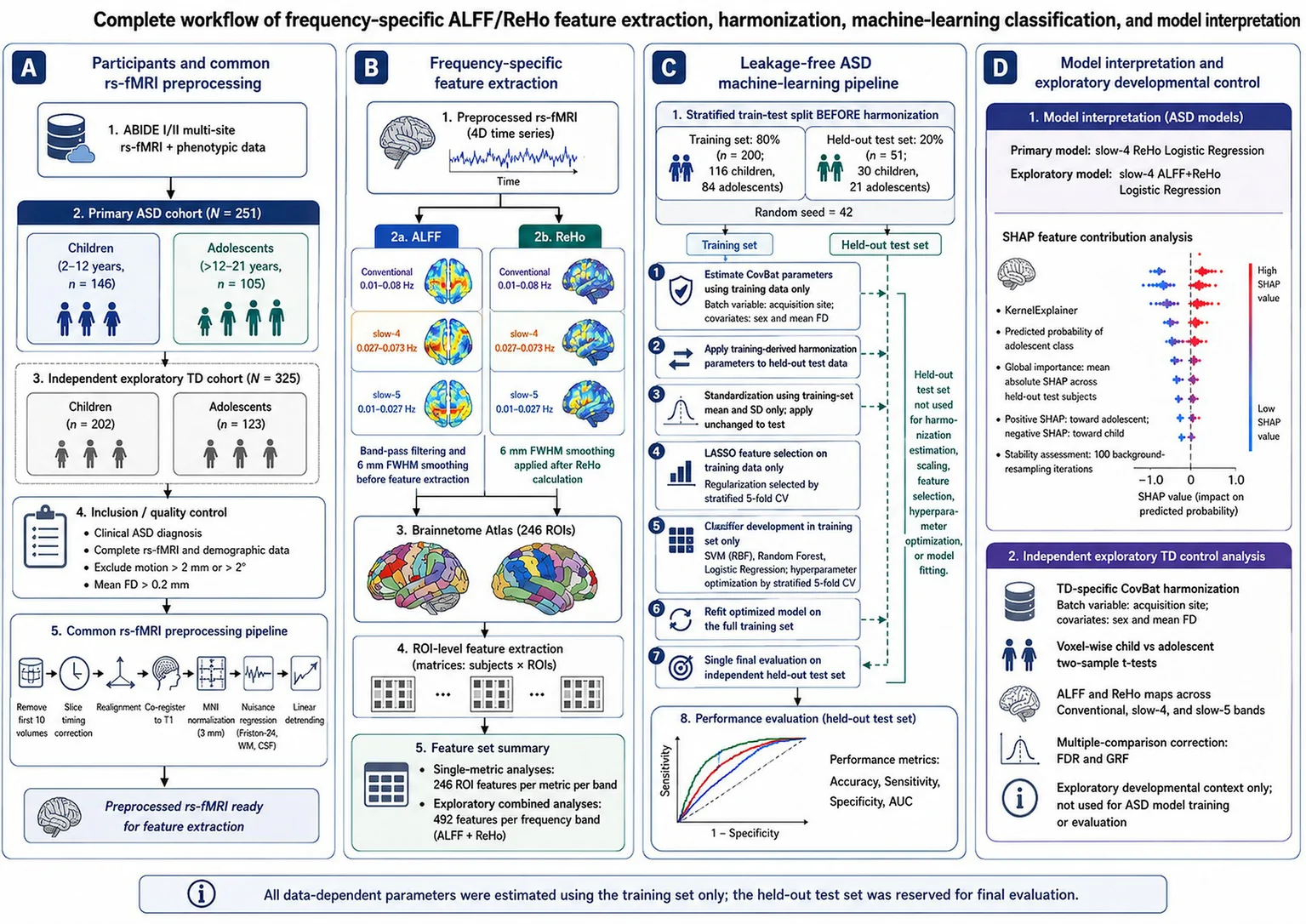
Complete workflow of frequency-specific ALFF/ReHo feature extraction, harmonization, machine-learning classification, and model interpretation **(a)** Participants and preprocessing. Multisite resting-state fMRI data were obtained from ABIDE I and II repositories. The ASD cohort was used for developmental-stage classification between children and adolescents, while the TD cohort was included as an exploratory developmental-stage control analysis. **(b)** Frequency-specific feature extraction. ALFF and ReHo maps were calculated across three frequency bands: Conventional (0.01–0.08 Hz), slow-4 (0.027–0.073 Hz), and slow-5 (0.01–0.027 Hz). ROI-level features were extracted using the Brainnetome Atlas containing 246 regions. **(c)** The ASD cohort was divided into a stratified training set (80%, *n* = 200) and a held-out test set (20%, *n* = 51) before CovBat harmonization, using a fixed random seed of 42. CovBat harmonization, feature scaling, LASSO feature selection, and classifier hyperparameter optimization were estimated using the training data only. The resulting training-derived transformations and fitted models were subsequently applied to the held-out test set for final evaluation. **(d)** Model interpretation and control analysis. SHAP analysis was performed for the primary slow-4 ReHo Logistic Regression model and the exploratory slow-4 ALFF+ReHo Logistic Regression model. TD developmental-stage control analysis was performed using TD-specific CovBat harmonization followed by corrected whole-brain statistical analysis.

SHAP values were computed using the final saved models after model training and held-out test evaluation to quantify feature-level contributions to model predictions. KernelExplainer was used to explain the predicted probability of the adolescent class. Global feature importance was defined as the mean absolute SHAP value across held-out test subjects. Positive SHAP values indicated contributions toward adolescent classification, whereas negative SHAP values indicated contributions toward child classification. Explanation stability was assessed using 100 background-resampling iterations. The overall study workflow, from rs-fMRI preprocessing through frequency-specific feature extraction, data harmonization, machine learning classification, and model interpretation, is summarized in Figure 1.

## 3 Results

### 3.1 Participant characteristics

A total of 251 ASD participants were included in the primary analysis, comprising 146 children and 105 adolescents. The two developmental-stage groups differed significantly in age (*p* < 0.001), as expected from the age-based grouping criteria. Sex distribution also differed between groups (*p* = 0.046), with a higher proportion of males among adolescents, whereas mean framewise displacement did not differ significantly (*p* = 0.604). Age, sex, and mean framewise displacement were included as covariates in subsequent harmonization procedures. The demographic and motion characteristics of the ASD cohort are summarized in Table 1.

**Table 1:** ASD demographic characteristics.

Characteristic	Children (*n* = 146)	Adolescents (*n* = 105)	Total (*n* = 251)	Statistic	*p*-value
Age (years), mean (SD)	9.10 (1.90)	14.98 (2.16)	11.56 (3.53)	t (249) = −22.83	<0.001
Sex, *n* (%)				χ^2^(1) = 3.97	0.046
Male	123 (84.2%)	98 (93.3%)	221 (88.0%)		
Female	23 (15.8%)	7 (6.7%)	30 (12.0%)		
Mean FD(mm),mean(SD)	0.094 (0.04)	0.091 (0.04)	0.093 (0.042)	t(249) = 0.52	0.604

FD, framewise displacement. Group comparisons were conducted using independent samples t-tests for continuous variables and the Pearson chi-square (χ2) test for categorical variables.

For the exploratory TD control analysis, 325 typically developing participants were included, comprising 202 children and 123 adolescents. TD children and adolescents differed in age (p < 0.001), mean framewise displacement (*p* = 0.0017), and sex distribution (*p* = 0.011). The TD cohort was analyzed independently from the ASD machine-learning pipeline and was used only to provide exploratory developmental context. Site-related variability in the TD cohort was addressed using TD-specific CovBat harmonization before whole-brain statistical testing. TD demographic and motion characteristics are summarized in Supplementary Table 1.

### 3.2 Feature selection

The number of ROI-level features selected by LASSO varied across different metrics and frequency bands and is presented in Table 2. In the single-metric analyses, 35 ALFF features were retained in the Conventional band, 42 in slow-4, and 31 in slow-5. For ReHo, 38 features were retained in the Conventional band, 53 in slow-4, and 23 in slow-5. The slow-4 ReHo feature set therefore had the largest selected single-metric feature subset. In the exploratory combined ALFF+ReHo analyses, the Conventional band retained 43 total features, including 27 ALFF and 16 ReHo features. The slow-4 combined feature set retained 51 total features, including 31 ALFF and 20 ReHo features. The slow-5 combined feature set retained 56 total features, including 30 ALFF and 26 ReHo features.

**Table 2:** LASSO feature selection summary.

Feature set	Frequency band	Initial features	Selected ALFF	Selected ReHo	Total selected
ALFF	Conventional	246	35	0	35
ReHo	Conventional	246	0	38	38
ALFF + ReHo	Conventional	492	27	16	43
ALFF	slow-4	246	42	0	42
ReHo	slow-4	246	0	53	53
ALFF+ReHo	slow-4	492	31	20	51
ALFF	slow-5	246	31	0	31
ReHo	slow-5	246	0	23	23
ALFF+ReHo	slow-5	492	30	26	56

### 3.3 Machine learning classification performance

**Figure 2:**
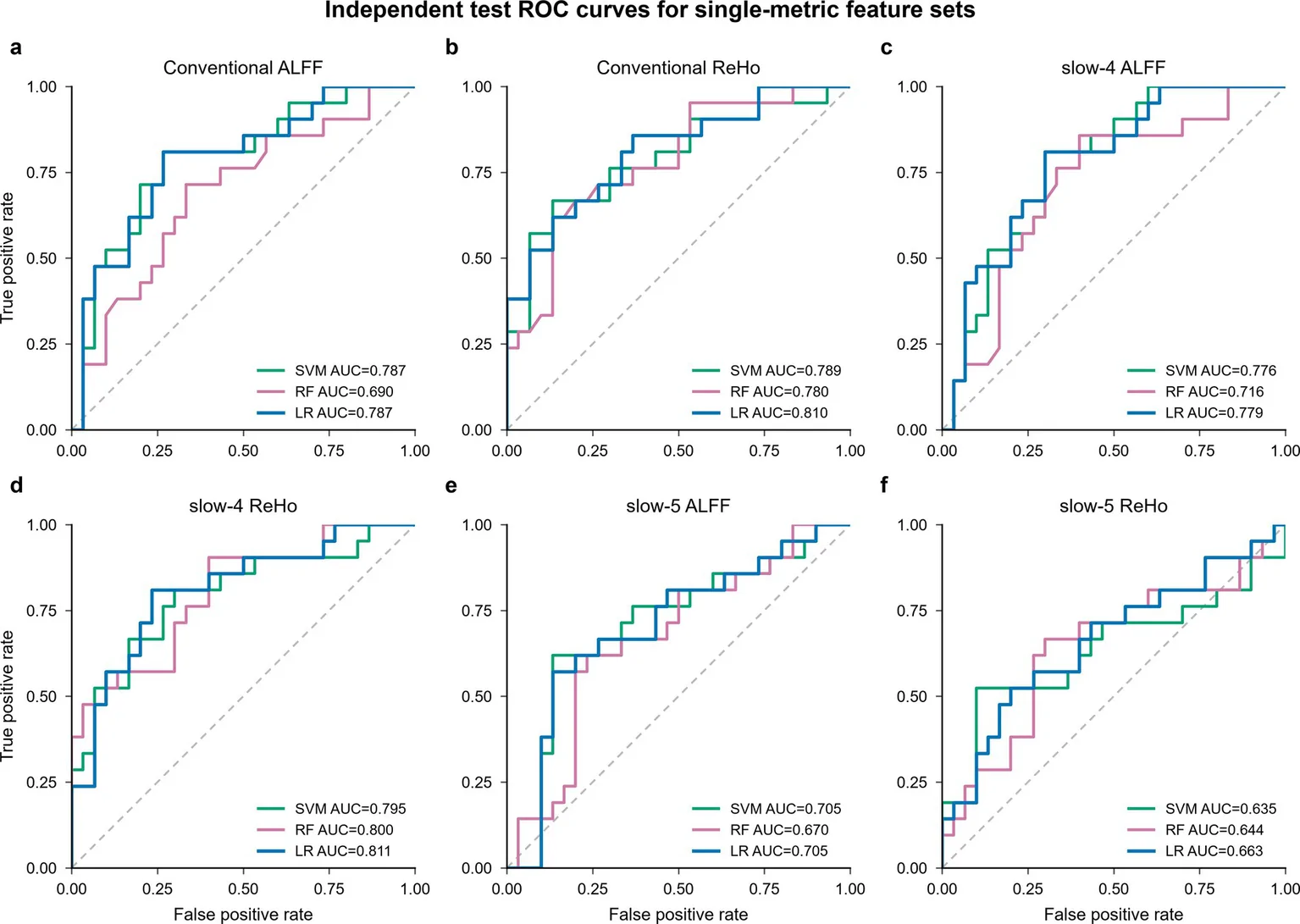
Receiver operating characteristic (ROC) curves for single-metric classification models on the independent held-out test set. ROC curves are shown for ALFF- and ReHo-based models across three frequency bands. **(a)** Conventional ALFF. **(b)** Conventional ReHo. **(c)** Slow-4 ALFF. **(d)** Slow-4 ReHo. **(e)** Slow-5 ALFF. **(f)** Slow-5 ReHo.

On the held-out ASD test set, the slow-4 ReHo Logistic Regression classifier achieved the best single-metric performance, with an AUC of 0.811, accuracy of 0.745, sensitivity of 0.571, and specificity of 0.867. The Conventional ReHo Logistic Regression model showed similar discrimination, with an AUC of 0.810 and accuracy of 0.706. In the exploratory combined-feature analyses, the slow-4 ALFF+ReHo Logistic Regression model achieved the highest overall AUC, with an AUC of 0.819, accuracy of 0.725, sensitivity of 0.762, and specificity of 0.700. The Conventional ALFF+ReHo Logistic Regression model also performed competitively, with an AUC of 0.806 and accuracy of 0.784. Across feature sets, the strongest held-out test performance was observed in the slow-4 and Conventional bands, whereas slow-5 models showed weaker discrimination. The highest-AUC slow-5 single-metric model reached an AUC of 0.705. Performance metrics for the highlighted models are summarized in Table 3, with the complete classifier comparison provided in Supplementary Table 2. ROC curves are shown in Figure 2 and Supplementary Figure 2, and AUC heatmaps are provided in Supplementary Figure 1.

**Table 3:** Best-performing classification models on the independent test set.

Model	Accuracy	Sensitivity	Specificity	AUC (95% CI)
slow-4 ReHo LR	0.745	0.571	0.867	0.811 (0.677–0.924)
slow-4 ALFF+ReHo LR	0.725	0.762	0.700	0.819 (0.674–0.935)

Sensitivity is the adolescent identification rate; specificity is the child identification rate. LR, logistic regression.

### 3.4 SHAP feature contributions of the primary slow-4 ReHo logistic regression model

**Figure 3:**
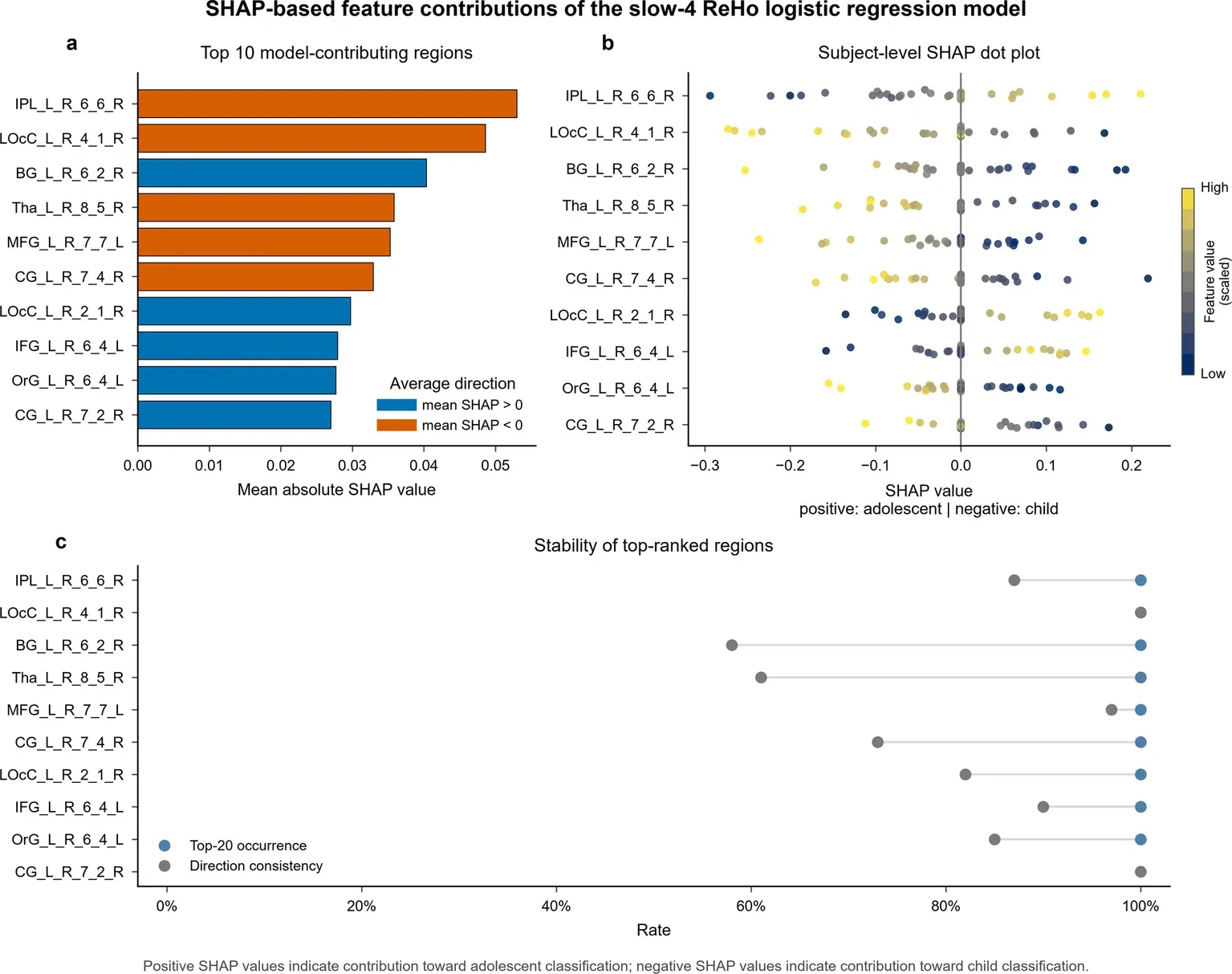
SHAP-based feature contributions of the primary slow-4 ReHo Logistic Regression model. **(a)** Global SHAP feature importance showing the top 10 model-contributing regions ranked by mean absolute SHAP value. **(b)** SHAP summary plot illustrating subject-level feature contributions. Positive SHAP values indicate contributions toward adolescent classification, whereas negative SHAP values indicate contributions toward child classification. **(c)** SHAP explanation stability across background-resampling iterations. Feature stability was summarized using top-ranked occurrence frequency and direction consistency. IPL, inferior parietal lobule; LOcC, lateral occipital cortex; IFG, inferior frontal gyrus; MFG, middle frontal gyrus; OrG, orbital gyrus; BG, basal ganglia; Tha, thalamus; CG, cingulate gyrus; STG, superior temporal gyrus; PhG, parahippocampal gyrus; L, left; R, right.

Features with the largest contributions to model predictions were mainly distributed across the inferior parietal lobule (IPL), lateral occipital cortex (LOcC), middle frontal gyrus (MFG), inferior frontal gyrus (IFG), basal ganglia, and thalamic regions (Figure 3; Table 4; Supplementary Table 3). Direction consistency was highest for several cortical features, whereas basal ganglia and thalamic features showed lower direction consistency across background-resampling iterations.

**Table 4:** Primary SHAP ranking for slow-4 ReHo logistic regression.

Rank	ROI	Mean absolute SHAP	Direction	Top-10 frequency	Direction consistency
1	IPL_L_R_6_6_R	0.053	Toward child	1.00	0.87
2	LOcC_L_R_4_1_R	0.049	Toward child	1.00	1.00
3	BG_L_R_6_2_R	0.040	Toward adolescent	1.00	0.58
4	Tha_L_R_8_5_R	0.036	Toward child	1.00	0.61
5	MFG_L_R_7_7_L	0.035	Toward child	1.00	0.97
6	CG_L_R_7_4_R	0.033	Toward child	0.98	0.73
7	LOcC_L_R_2_1_R	0.030	Toward adolescent	0.97	0.82
8	IFG_L_R_6_4_L	0.028	Toward adolescent	0.82	0.90
9	OrG_L_R_6_4_L	0.028	Toward adolescent	0.87	0.85
10	CG_L_R_7_2_R	0.027	Toward adolescent	0.17	1.00

IPL, inferior parietal lobule; LOcC, lateral occipital cortex; BG, basal ganglia; Tha, thalamus; MFG, middle frontal gyrus; CG, cingulate gyrus; IFG, inferior frontal gyrus; OrG, orbital gyrus; L, left hemisphere; R, right hemisphere. Mean absolute SHAP represents global feature importance across held-out test participants. Direction was determined from the mean signed SHAP value, with positive contributions indicating adolescent classification and negative contributions indicating child classification. Top-10 frequency represents the proportion of 100 background-resampling iterations in which a feature ranked among the 10 most important features. Direction consistency represents the proportion of iterations in which the feature retained the same contribution direction.

**Figure 4:**
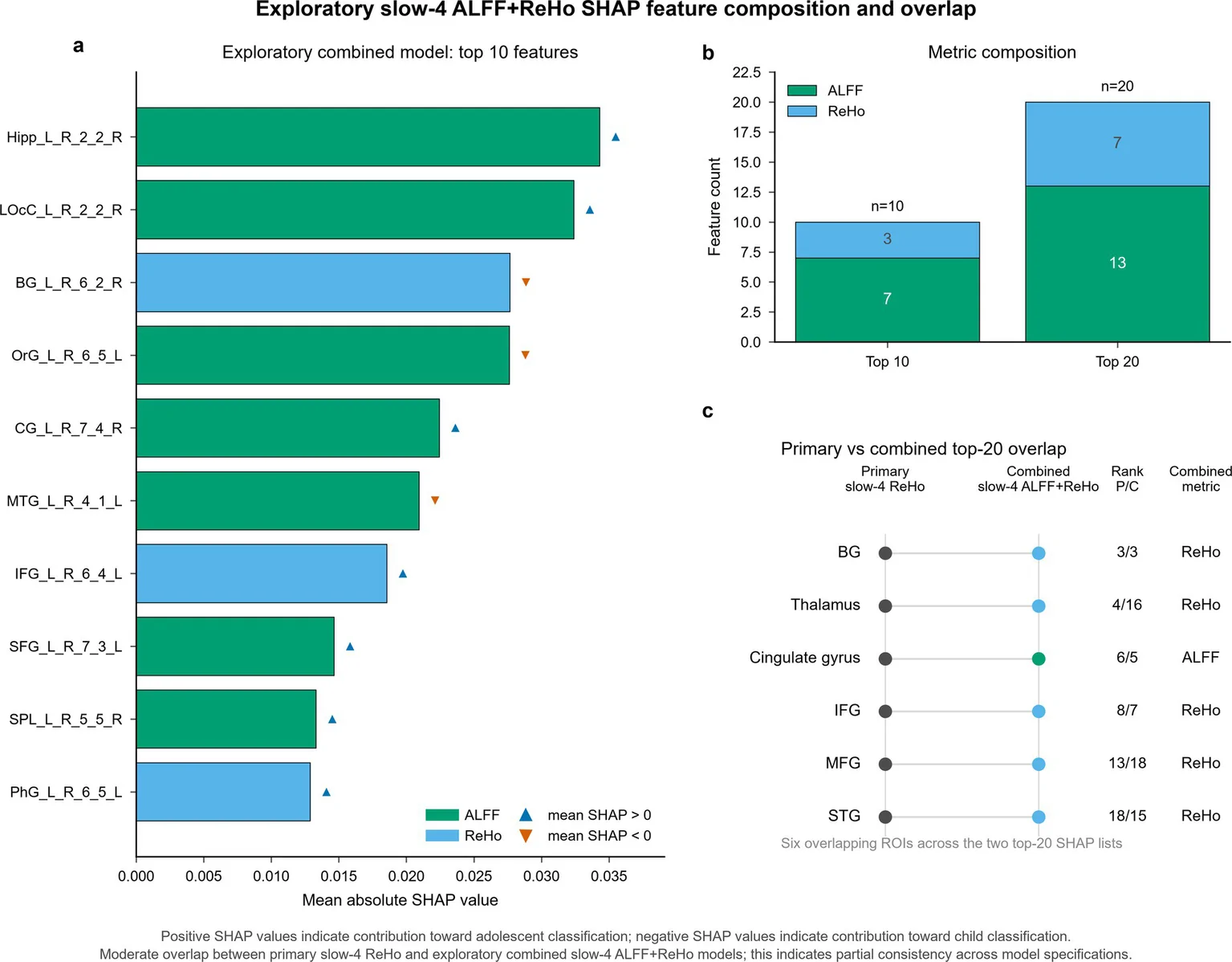
Exploratory SHAP analysis of the combined slow-4 ALFF+ReHo Logistic Regression model and overlap with the primary model **(a)** Global SHAP importance of the top 10 features in the exploratory combined slow-4 ALFF+ReHo model. Features are colored according to imaging metric (ALFF or ReHo). **(b)** Contribution composition of ALFF and ReHo features among the top-ranked combined-model features. **(c)** Comparison of the top-20 SHAP regions between the primary slow-4 ReHo model and the exploratory combined slow-4 ALFF+ReHo model. Shared regions indicate partial consistency across model formulations. ALFF, amplitude of low-frequency fluctuation; ReHo, regional homogeneity; IPL, inferior parietal lobule; LOcC, lateral occipital cortex; IFG, inferior frontal gyrus; MFG, middle frontal gyrus; BG, basal ganglia; Tha, thalamus; CG, cingulate gyrus; STG, superior temporal gyrus.

Exploratory SHAP results for the combined slow-4 ALFF+ReHo Logistic Regression model, together with the overlap between the single-metric and combined-feature models, are summarized in Figure 4 and Supplementary Tables 4, 5.

### 3.5 Exploratory TD control analysis

The exploratory TD control analysis was performed to examine whether developmental-stage-related differences were also detectable in typical development. TD imaging data were independently harmonized using TD-specific CovBat. Voxel-wise two-sample t-tests were then performed between TD children and TD adolescents for each ALFF and ReHo frequency-band map, with sex, and mean FD included as covariates. Across all three frequency bands, Conventional, slow-4, and slow-5, no significant child-adolescent differences were detected for either ALFF or ReHo after FDR or GRF correction (Supplementary Table 6).

## 4 Discussion

This study investigated whether frequency-specific resting-state ALFF and ReHo features contain multivariate information associated with age-defined developmental stages (children vs. adolescents) within ASD. Using a rigorous pipeline involving pre-harmonization train-test splitting, CovBat harmonization, LASSO feature selection, and held-out test evaluation, we found that frequency-specific resting-state features provided meaningful discrimination between developmental stages. Across the evaluated models, the slow-4 and Conventional frequency bands consistently outperformed the slow-5 band, with the slow-4 ReHo Logistic Regression model achieving the strongest performance among the single-metric analyses (AUC = 0.811). The exploratory combined slow-4 ALFF+ReHo Logistic Regression model further achieved the highest overall classification performance (AUC = 0.819), suggesting that local functional synchronization and regional spontaneous activity may provide complementary information for characterizing developmental-stage-related functional variation within ASD. Together, these findings demonstrate that frequency-specific intrinsic brain activity contains measurable multivariate information associated with developmental stage and further highlight the utility of studying developmental heterogeneity in multisite neuroimaging datasets.

A principal finding of this study is that the slow-4 and Conventional frequency bands consistently demonstrated superior classification performance relative to the slow-5 band across both ALFF and ReHo features. Although resting-state BOLD oscillations within the conventional low-frequency range (0.01–0.08 Hz) have long been considered physiologically meaningful, accumulating evidence indicates that different frequency sub-bands capture distinct aspects of spontaneous neural activity (Yu-Feng et al., 2007; Zuo et al., 2010). In particular, slow-4 oscillations (0.027–0.073 Hz) have been associated with stronger cortical functional organization and greater sensitivity to gray matter physiological processes than slower oscillatory components, whereas slow-5 oscillations may preferentially reflect large-scale cortical integration and long-range synchronization. Consistent with this framework, the present findings suggest that developmental-stage-related functional differences within ASD are more prominently represented in the higher portion of the conventional low-frequency spectrum.

Although the physiological basis underlying this observation cannot be determined from the present cross-sectional analysis, previous developmental resting-state studies have likewise demonstrated age-dependent alterations in spontaneous brain activity in ASD, including atypical developmental trajectories of ALFF within default-mode and frontal regions, supporting the importance of considering developmental stage when characterizing intrinsic brain function (Guo et al., 2017; Hull et al., 2017). The superior performance of the slow-4 ReHo model is consistent with previous developmental studies demonstrating that local functional synchronization exhibits age-dependent alterations in ASD, suggesting that maturation of intrinsic local connectivity differs across developmental stages (Dajani and Uddin, 2016; Hull et al., 2017).

To further characterize the multivariate patterns underlying developmental-stage classification, SHAP analysis demonstrated a distributed pattern of model-contributing regions in the slow-4 ReHo Logistic Regression model. Rather than identifying isolated biomarker regions, SHAP highlighted a distributed set of cortical and subcortical regions whose collective contributions supported classification between children and adolescents within ASD. This distributed pattern is consistent with the view that brain maturation reflects coordinated reorganization across large-scale functional systems rather than localized regional changes alone (Casey et al., 2005; Fair et al., 2009). Importantly, SHAP quantifies each feature’s contribution to model predictions and should therefore be interpreted as identifying regions that contribute to model predictions rather than as direct evidence of neurobiological mechanisms or ASD-specific developmental abnormalities. Among all identified features, the inferior parietal lobule exhibited the largest contribution to developmental-stage classification. The IPL is a major component of the frontoparietal control network and plays a central role in attentional control, multisensory integration, cognitive flexibility, and higher-order executive processing (Numssen et al., 2021). Moreover, the prominence of IPL features in the present model suggests that the maturation of frontoparietal systems substantially contributes to the patterns that distinguish developmental stages in ASD.

The lateral occipital cortex also demonstrated strong and highly consistent SHAP contributions. This region is classically associated with higher-order visual object representation and perceptual integration (Grill-Spector et al., 2001). Beyond visual object recognition, however, lateral occipital regions interact extensively with temporal and parietal association cortices to support visually guided attention, social perception, and the integration of complex sensory information. Altered functional organization of occipito-temporal systems has frequently been reported in ASD, particularly during visual processing, face perception, and social cognition tasks.

Several frontal regions, including the IFG, MFG, and orbital frontal cortex, consistently ranked among the most influential features. The IFG has well-established roles in inhibitory control (Aron et al., 2004), language-related processing control (Friederici, 2011), and broader cognitive control functions. Meanwhile, the MFG forms an important component of the dorsolateral prefrontal cortex and contributes to executive control, working memory, cognitive flexibility, and goal-directed behavior. Neurodevelopmental studies have consistently shown that prefrontal regions exhibit prolonged maturation throughout adolescence, reflecting continued refinement of executive-control systems and increasing efficiency of large-scale cognitive networks (Casey et al., 2005; Luna et al., 2010). The repeated appearance of frontal regions across both the primary slow-4 ReHo model and the exploratory combined ALFF+ReHo model therefore likely reflects maturation of higher-order executive systems rather than isolated regional alterations. Importantly, these findings are also broadly consistent with previous neuroimaging studies reporting atypical frontal functional organization in ASD, particularly within networks supporting executive function and cognitive control (Hull et al., 2017).

Subcortical structures also contributed substantially to model predictions, particularly the basal ganglia and thalamus. Previous structural and functional neuroimaging studies have implicated atypical maturation of these circuits in ASD, although reported findings remain heterogeneous across cohorts and developmental stages (Langen et al., 2011). In the present study, basal ganglia and thalamic features were consistently identified among the highest-ranked SHAP contributors and overlapped between the primary ReHo model and the exploratory combined model, suggesting that cortico-subcortical functional organization contributes to developmental-stage classification irrespective of the resting-state metric used. However, these subcortical features demonstrated lower direction consistency across background-resampling iterations than several cortical regions, indicating greater variability in their contribution to model predictions. The exploratory combined ALFF+ReHo model additionally identified hippocampal and parahippocampal regions among the highest-ranking ALFF-derived contributors. The parahippocampal gyrus is involved in contextual representation, episodic memory, and scene processing and serves as an important interface between medial temporal memory systems and distributed association cortices (Aminoff et al., 2013). Although these limbic regions were less prominent in the primary ReHo model, their emergence in the combined analysis suggests that regional spontaneous activity amplitude may capture additional aspects of developmental-stage-related functional organization beyond those represented by local synchronization alone.

Comparison between the primary slow-4 ReHo model and the exploratory combined slow-4 ALFF+ReHo model demonstrated both convergence and divergence in the anatomical distribution of influential features. Six regional groups in both the top 20 lists, including the basal ganglia, thalamus, cingulate cortex, inferior frontal gyrus, middle frontal gyrus, and superior temporal gyrus, were represented among the highest-ranking contributors in both models. This overlap suggests that these regions provide robust information for developmental-stage classification regardless of whether spontaneous activity is characterized primarily through local synchronization or regional amplitude. At the same time, the combined model emphasized additional ALFF-derived contributions within occipital, temporal, limbic, and frontal association regions, indicating that ALFF and ReHo capture partly overlapping yet distinct aspects of intrinsic functional organization. These observations are consistent with previous resting-state studies demonstrating that ALFF and ReHo characterize complementary physiological properties of spontaneous BOLD activity and therefore provide nonredundant information regarding brain functional architecture (Biswal et al., 2010).

The exploratory TD control analysis provided developmental context for the ASD classification findings. In the TD cohort, voxel-wise two-sample t-tests with sex and mean FD as covariates did not reveal significant child-adolescent differences in any ALFF or ReHo frequency-band map after FDR or GRF correction. This result indicates that, under the present sample and statistical-thresholding framework, comparable corrected whole-brain developmental-stage differences were not detected in the TD cohort. The TD analysis therefore provides a useful contextual comparison for the ASD findings while leaving diagnosis-by-developmental-stage interactions for future studies.

Several limitations should be considered. First, the models distinguished developmental stages within ASD and were not designed to diagnose ASD or predict individual clinical outcomes. Second, although the held-out test set provides an internal estimate of generalization under the present pipeline, external validation in independent cohorts is still needed. Third, although CovBat was used to reduce site-related effects and age, sex, and mean FD were considered in harmonization and control analyses, residual confounding related to site distribution, site-by-age effects, sex imbalance, motion, and clinical heterogeneity cannot be fully excluded. Fourth, the TD analysis was exploratory and did not directly test a formal diagnosis-by-developmental-stage interaction. Fifth, SHAP analysis provides model attribution rather than causal inference. Therefore, SHAP-derived regions should be interpreted as model-contributing candidates rather than causal neurobiological mechanisms. Finally, the cross-sectional design limits conclusions about individual developmental trajectories; longitudinal studies are needed to determine whether these features track within-person developmental change.

## 5 Conclusion

The present study demonstrates that frequency-specific resting-state ALFF and ReHo features contain meaningful multivariate information capable of distinguishing children from adolescents within an ASD cohort. Across the evaluated feature sets, the slow-4 and Conventional frequency bands consistently outperformed the slow-5 band, with slow-4 ReHo providing the strongest single-metric classification performance. SHAP analysis indicated that developmental-stage classification was supported by distributed contributions from frontoparietal, visual association, frontal, cingulate, and cortico-subcortical systems rather than isolated regional effects, whereas the exploratory combined ALFF+ReHo analysis suggested that regional spontaneous activity amplitude and local functional synchronization provide partially complementary representations of intrinsic brain function. Although these findings do not establish causal neurobiological mechanisms or ASD-specific developmental trajectories, they support the utility of frequency-specific resting-state metrics as candidate indicators of developmental-stage-related functional heterogeneity within ASD. More broadly, the study provides a rigorously evaluated machine-learning framework for investigating neurodevelopmental variation in multisite resting-state fMRI datasets and establishes a foundation for future longitudinal and externally validated investigations of brain maturation in autism.

## Data Availability

Publicly available datasets were analyzed in this study. This data can be found here: Brain Imaging Data Exchange (ABIDE I and II) repositories (http://fcon_1000.projects.nitrc.org/indi/abide/).
