# Supplementary material for: Machine-learning classification of children and adolescents with ASD using resting-state frequency-specific intrinsic activity
Source: Front Neurosci. 2026 Aug 7;20:1892288. doi: 10.3389/fnins.2026.1892288 (PMC13504999; doi:10.3389/fnins.2026.1892288)
Supplement: Supplementary file 1 [file Supplementary_file_1.docx]

**Supplementary Materials**

**Supplementary Tables**

Supplementary Table 1 TD demographic characteristics.

| **Characteristic** | **TD children (n=202)** | **TD adolescents (n=123)** | **Overall (n=325)** | **Statistic** | **p-value** |
| --- | --- | --- | --- | --- | --- |
| Age, years, mean (SD) | 9.81 (1.39) | 14.60 (2.23) | 11.62 (2.91) | t = −21.75 | <0.001 |
| Sex, Male, n (%) | 125 (61.9%) | 93 (75.6%) | 218 (67.1%) | χ² = 6.50 | 0.011 |
| Sex, Female, n (%) | 77 (38.1%) | 30 (24.4%) | 107 (32.9%) |  |  |
| Mean FD, mm, mean (SD) | 0.0829 (0.0370) | 0.0707 (0.0314) | 0.0783 (0.0354) | t = 2.99 | 0.003 |

Note. FD, Framewise Displacement. Group comparisons were conducted using independent samples t-tests for continuous variables and the Pearson chi-square (χ2) test for categorical variables.

Supplementary Table 2 Complete held-out ASD test-set classification performance across all models and feature sets.

| **Feature set** | **Frequency band** | **Model** | **Accuracy** | **Sensitivity** | **Specificity** | **AUC (95% CI)** |
| --- | --- | --- | --- | --- | --- | --- |
| ALFF | Conventional | Logistic Regression | 0.725 | 0.714 | 0.733 | 0.787 (0.644-0.910) |
| ALFF | Conventional | SVM | 0.745 | 0.667 | 0.800 | 0.787 (0.652-0.909) |
| ALFF | Conventional | Random Forest | 0.627 | 0.429 | 0.767 | 0.690 (0.535-0.834) |
| ALFF | slow-4 | Logistic Regression | 0.745 | 0.810 | 0.700 | 0.779 (0.637-0.888) |
| ALFF | slow-4 | SVM | 0.686 | 0.571 | 0.767 | 0.776 (0.630-0.884) |
| ALFF | slow-4 | Random Forest | 0.686 | 0.571 | 0.767 | 0.716 (0.557-0.850) |
| ALFF | slow-5 | Logistic Regression | 0.706 | 0.619 | 0.767 | 0.705 (0.554-0.849) |
| ALFF | slow-5 | SVM | 0.725 | 0.619 | 0.800 | 0.705 (0.560-0.853) |
| ALFF | slow-5 | Random Forest | 0.647 | 0.619 | 0.667 | 0.670 (0.513-0.814) |
| ReHo | Conventional | Logistic Regression | 0.706 | 0.714 | 0.700 | 0.810 (0.682-0.922) |
| ReHo | Conventional | SVM | 0.706 | 0.667 | 0.733 | 0.789 (0.646-0.908) |
| ReHo | Conventional | Random Forest | 0.745 | 0.619 | 0.833 | 0.780 (0.647-0.898) |
| ReHo | slow-4 | Logistic Regression | 0.745 | 0.571 | 0.867 | 0.811 (0.677-0.924) |
| ReHo | slow-4 | SVM | 0.765 | 0.667 | 0.833 | 0.795 (0.647-0.917) |
| ReHo | slow-4 | Random Forest | 0.745 | 0.571 | 0.867 | 0.800 (0.674-0.909) |
| ReHo | slow-5 | Logistic Regression | 0.627 | 0.714 | 0.567 | 0.663 (0.506-0.832) |
| ReHo | slow-5 | SVM | 0.627 | 0.524 | 0.700 | 0.635 (0.464-0.815) |
| ReHo | slow-5 | Random Forest | 0.686 | 0.619 | 0.733 | 0.644 (0.478-0.821) |
| ALFF+ReHo | Conventional | Logistic Regression | 0.784 | 0.762 | 0.800 | 0.806 (0.669-0.927) |
| ALFF+ReHo | Conventional | SVM | 0.804 | 0.762 | 0.833 | 0.768 (0.625-0.898) |
| ALFF+ReHo | Conventional | Random Forest | 0.745 | 0.762 | 0.733 | 0.766 (0.606-0.917) |
| ALFF+ReHo | slow-4 | Logistic Regression | 0.725 | 0.762 | 0.700 | 0.819 (0.674-0.935) |
| ALFF+ReHo | slow-4 | SVM | 0.824 | 0.714 | 0.900 | 0.798 (0.652-0.926) |
| ALFF+ReHo | slow-4 | Random Forest | 0.765 | 0.667 | 0.833 | 0.746 (0.595-0.879) |
| ALFF+ReHo | slow-5 | Logistic Regression | 0.647 | 0.571 | 0.700 | 0.702 (0.553-0.862) |
| ALFF+ReHo | slow-5 | SVM | 0.588 | 0.429 | 0.700 | 0.656 (0.508-0.813) |
| ALFF+ReHo | slow-5 | Random Forest | 0.608 | 0.429 | 0.733 | 0.639 (0.485-0.797) |

Note. Sensitivity is the adolescent identification rate; specificity is the child identification rate. ASD, autism spectrum disorder; ALFF, amplitude of low-frequency fluctuation; ReHo, regional homogeneity; SVM, support vector machine; LR, Logistic Regression; AUC, area under the receiver operating characteristic curve; CI, confidence interval.

Supplementary Table 3 Full SHAP feature ranking for the primary slow-4 ReHo Logistic Regression model.

| **Rank** | **ROI** | **Functional system** | **Mean absolute SHAP** | **Direction** | **Top-10 frequency** | **Direction consistency** |
| --- | --- | --- | --- | --- | --- | --- |
| 1 | IPL_L_R_6_6_R | parietal/DMN-frontoparietal | 0.053 | Toward child | 1.00 | 0.87 |
| 2 | LOcC_L_R_4_1_R | temporal/visual-language or social-perceptual | 0.049 | Toward child | 1.00 | 1.00 |
| 3 | BG_L_R_6_2_R | subcortical developmental-control | 0.040 | Toward adolescent | 1.00 | 0.58 |
| 4 | Tha_L_R_8_5_R | subcortical developmental-control | 0.036 | Toward child | 1.00 | 0.61 |
| 5 | MFG_L_R_7_7_L | frontal/frontoparietal-control | 0.035 | Toward child | 1.00 | 0.97 |
| 6 | CG_L_R_7_4_R | Not specified | 0.033 | Toward child | 0.98 | 0.73 |
| 7 | LOcC_L_R_2_1_R | temporal/visual-language or social-perceptual | 0.030 | Toward adolescent | 0.97 | 0.82 |
| 8 | IFG_L_R_6_4_L | frontal/frontoparietal-control | 0.028 | Toward adolescent | 0.82 | 0.90 |
| 9 | OrG_L_R_6_4_L | frontal/frontoparietal-control | 0.028 | Toward adolescent | 0.87 | 0.85 |
| 10 | CG_L_R_7_2_R | Not specified | 0.027 | Toward adolescent | 0.17 | 1.00 |
| 11 | MFG_L_R_7_7_R | frontal/frontoparietal-control | 0.026 | Toward adolescent | 0.75 | 0.52 |
| 12 | CG_L_R_7_1_L | Not specified | 0.024 | Toward adolescent | 0.12 | 0.63 |
| 13 | MFG_L_R_7_3_L | frontal/frontoparietal-control | 0.023 | Toward child | 0.18 | 0.91 |
| 14 | STG_L_R_6_4_R | temporal/visual-language or social-perceptual | 0.023 | Toward child | 0.00 | 0.89 |
| 15 | PhG_L_R_6_6_L | limbic/memory-related | 0.022 | Toward child | 0.13 | 0.99 |
| 16 | CG_L_R_7_3_L | Not specified | 0.022 | Toward child | 0.00 | 0.00 |
| 17 | MFG_L_R_7_6_L | frontal/frontoparietal-control | 0.018 | Toward child | 0.00 | 0.65 |
| 18 | STG_L_R_6_5_R | temporal/visual-language or social-perceptual | 0.016 | Toward child | 0.00 | 0.72 |
| 19 | ITG_L_R_7_7_R | temporal/visual-language or social-perceptual | 0.016 | Toward child | 0.01 | 0.98 |
| 20 | PCun_L_R_4_2_R | parietal/DMN-frontoparietal | 0.014 | Toward adolescent | 0.00 | 0.93 |

Note. Primary model: slow-4 ReHo Logistic Regression. Positive SHAP values contribute toward adolescent classification, whereas negative SHAP values contribute toward child classification. SHAP, Shapley Additive Explanations.

Supplementary Table 4 Full SHAP feature ranking for the exploratory slow-4 ALFF+ReHo Logistic Regression model.

| **Rank** | **Metric** | **ROI** | **Functional system** | **Mean absolute SHAP** | **Direction** | **Top-10 frequency** | **Direction consistency** |
| --- | --- | --- | --- | --- | --- | --- | --- |
| 1 | ALFF | Hipp_L_R_2_2_R | limbic/memory-related | 0.034 | Toward adolescent | 1.00 | 1.00 |
| 2 | ALFF | LOcC_L_R_2_2_R | temporal/visual-language or social-perceptual | 0.032 | Toward adolescent | 1.00 | 0.92 |
| 3 | ReHo | BG_L_R_6_2_R | subcortical developmental-control | 0.028 | Toward child | 1.00 | 0.84 |
| 4 | ALFF | OrG_L_R_6_5_L | frontal/frontoparietal-control | 0.028 | Toward child | 1.00 | 0.93 |
| 5 | ALFF | CG_L_R_7_4_R | Not specified | 0.022 | Toward adolescent | 1.00 | 0.86 |
| 6 | ALFF | MTG_L_R_4_1_L | temporal/visual-language or social-perceptual | 0.021 | Toward child | 0.99 | 0.99 |
| 7 | ReHo | IFG_L_R_6_4_L | frontal/frontoparietal-control | 0.019 | Toward adolescent | 1.00 | 0.91 |
| 8 | ALFF | SFG_L_R_7_3_L | frontal/frontoparietal-control | 0.015 | Toward adolescent | 0.59 | 0.98 |
| 9 | ALFF | SPL_L_R_5_5_R | parietal/DMN-frontoparietal | 0.013 | Toward adolescent | 0.73 | 0.84 |
| 10 | ReHo | PhG_L_R_6_5_L | limbic/memory-related | 0.013 | Toward adolescent | 0.40 | 0.59 |
| 11 | ALFF | PhG_L_R_6_4_L | limbic/memory-related | 0.013 | Toward child | 0.47 | 0.92 |
| 12 | ReHo | OrG_L_R_6_4_R | frontal/frontoparietal-control | 0.012 | Toward adolescent | 0.05 | 0.84 |
| 13 | ALFF | INS_L_R_6_5_R | Not specified | 0.012 | Toward child | 0.74 | 0.78 |
| 14 | ALFF | pSTS_L_R_2_2_R | Not specified | 0.011 | Toward child | 0.03 | 0.72 |
| 15 | ReHo | STG_L_R_6_5_R | temporal/visual-language or social-perceptual | 0.011 | Toward child | 0.00 | 0.68 |
| 16 | ReHo | Tha_L_R_8_5_R | subcortical developmental-control | 0.010 | Toward child | 0.00 | 1.00 |
| 17 | ALFF | PCun_L_R_4_2_L | parietal/DMN-frontoparietal | 0.009 | Toward child | 0.00 | 0.70 |
| 18 | ReHo | MFG_L_R_7_3_L | frontal/frontoparietal-control | 0.008 | Toward child | 0.00 | 0.79 |
| 19 | ALFF | CG_L_R_7_6_L | Not specified | 0.008 | Toward adolescent | 0.00 | 0.96 |
| 20 | ALFF | IFG_L_R_6_6_L | frontal/frontoparietal-control | 0.008 | Toward child | 0.00 | 0.61 |

Note. Exploratory model: slow-4 ALFF+ReHo Logistic Regression. The Metric column indicates whether each feature came from ALFF or ReHo. Positive SHAP values contribute toward adolescent classification, whereas negative SHAP values contribute toward child classification.

Supplementary Table 5 Overlap between primary and exploratory combined-model SHAP rankings.

| **ROI** | **Primary top-20** | **Combined top-20** | **Primary rank** | **Combined rank** | **Primary mean absolute SHAP** | **Combined mean absolute SHAP** | **Combined metric** | **Status** |
| --- | --- | --- | --- | --- | --- | --- | --- | --- |
| BG_L_R_6_2_R | Yes | Yes | 3 | 3 | 0.040 | 0.028 | ReHo | Overlap |
| Tha_L_R_8_5_R | Yes | Yes | 4 | 16 | 0.036 | 0.010 | ReHo | Overlap |
| CG_L_R_7_4_R | Yes | Yes | 6 | 5 | 0.033 | 0.022 | ALFF | Overlap |
| IFG_L_R_6_4_L | Yes | Yes | 8 | 7 | 0.028 | 0.019 | ReHo | Overlap |
| MFG_L_R_7_3_L | Yes | Yes | 13 | 18 | 0.023 | 0.008 | ReHo | Overlap |
| STG_L_R_6_5_R | Yes | Yes | 18 | 15 | 0.016 | 0.011 | ReHo | Overlap |
| IPL_L_R_6_6_R | Yes | No | 1 | NA | 0.053 | NA | NA | Primary only |
| LOcC_L_R_4_1_R | Yes | No | 2 | NA | 0.049 | NA | NA | Primary only |
| MFG_L_R_7_7_L | Yes | No | 5 | NA | 0.035 | NA | NA | Primary only |
| LOcC_L_R_2_1_R | Yes | No | 7 | NA | 0.030 | NA | NA | Primary only |
| OrG_L_R_6_4_L | Yes | No | 9 | NA | 0.028 | NA | NA | Primary only |
| CG_L_R_7_2_R | Yes | No | 10 | NA | 0.027 | NA | NA | Primary only |
| MFG_L_R_7_7_R | Yes | No | 11 | NA | 0.026 | NA | NA | Primary only |
| CG_L_R_7_1_L | Yes | No | 12 | NA | 0.024 | NA | NA | Primary only |
| STG_L_R_6_4_R | Yes | No | 14 | NA | 0.023 | NA | NA | Primary only |
| PhG_L_R_6_6_L | Yes | No | 15 | NA | 0.022 | NA | NA | Primary only |
| CG_L_R_7_3_L | Yes | No | 16 | NA | 0.022 | NA | NA | Primary only |
| MFG_L_R_7_6_L | Yes | No | 17 | NA | 0.018 | NA | NA | Primary only |
| ITG_L_R_7_7_R | Yes | No | 19 | NA | 0.016 | NA | NA | Primary only |
| PCun_L_R_4_2_R | Yes | No | 20 | NA | 0.014 | NA | NA | Primary only |
| Hipp_L_R_2_2_R | No | Yes | NA | 1 | NA | 0.034 | ALFF | Combined only |
| LOcC_L_R_2_2_R | No | Yes | NA | 2 | NA | 0.032 | ALFF | Combined only |
| OrG_L_R_6_5_L | No | Yes | NA | 4 | NA | 0.028 | ALFF | Combined only |
| MTG_L_R_4_1_L | No | Yes | NA | 6 | NA | 0.021 | ALFF | Combined only |
| SFG_L_R_7_3_L | No | Yes | NA | 8 | NA | 0.015 | ALFF | Combined only |
| SPL_L_R_5_5_R | No | Yes | NA | 9 | NA | 0.013 | ALFF | Combined only |
| PhG_L_R_6_5_L | No | Yes | NA | 10 | NA | 0.013 | ReHo | Combined only |
| PhG_L_R_6_4_L | No | Yes | NA | 11 | NA | 0.013 | ALFF | Combined only |
| OrG_L_R_6_4_R | No | Yes | NA | 12 | NA | 0.012 | ReHo | Combined only |
| INS_L_R_6_5_R | No | Yes | NA | 13 | NA | 0.012 | ALFF | Combined only |
| pSTS_L_R_2_2_R | No | Yes | NA | 14 | NA | 0.011 | ALFF | Combined only |
| PCun_L_R_4_2_L | No | Yes | NA | 17 | NA | 0.009 | ALFF | Combined only |
| CG_L_R_7_6_L | No | Yes | NA | 19 | NA | 0.008 | ALFF | Combined only |
| IFG_L_R_6_6_L | No | Yes | NA | 20 | NA | 0.008 | ALFF | Combined only |

Note. Overlap compares top-20 ROIs between the primary slow-4 ReHo Logistic Regression model and the exploratory slow-4 ALFF+ReHo Logistic Regression model. NA indicates not applicable because the ROI was absent from the corresponding top-20 list.

Supplementary Table 6 Exploratory TD developmental-stage control analysis results.

| **Metric** | **Frequency band** | **N** | **Comparison** | **Covariates** | **FDR-corrected result** | **GRF-corrected result** |
| --- | --- | --- | --- | --- | --- | --- |
| ALFF | Conventional | 325 | TD children vs adolescents | Sex and mean FD | None detected | None detected |
| ALFF | slow-4 | 325 | TD children vs adolescents | Sex and mean FD | None detected | None detected |
| ALFF | slow-5 | 325 | TD children vs adolescents | Sex and mean FD | None detected | None detected |
| ReHo | Conventional | 325 | TD children vs adolescents | Sex and mean FD | None detected | None detected |
| ReHo | slow-4 | 325 | TD children vs adolescents | Sex and mean FD | None detected | None detected |
| ReHo | slow-5 | 325 | TD children vs adolescents | Sex and mean FD | None detected | None detected |

Note. None detected indicates no corrected TD child-adolescent difference after TD-specific CovBat harmonization and covariate-adjusted whole-brain GLM testing. FDR, false discovery rate; GRF, Gaussian random field.

**Supplementary Figures**

Supplementary Figure 1 Model-performance AUC heatmaps. Heatmaps summarize held-out ASD test-set AUC values across evaluated feature sets, frequency bands, and classifiers. Panel a shows the training 5-fold cross-validation AUC heatmap, and panel b shows the independent-test AUC heatmap.

Supplementary Figure 2 Held-out ASD test-set ROC curves for exploratory combined models. ROC curves are shown for exploratory ALFF+ReHo models across the Conventional, slow-4, and slow-5 frequency bands. Panel a shows ROC curves for the Conventional ALFF+ReHo feature set, panel b shows ROC curves for the slow-4 ALFF+ReHo feature set, and panel c shows ROC curves for the slow-5 ALFF+ReHo feature set.

Abbreviations: ASD = autism spectrum disorder; TD = typically developing; ALFF = amplitude of low-frequency fluctuation; ReHo = regional homogeneity; SHAP = Shapley Additive Explanations; FD = framewise displacement; LR = Logistic Regression.
